# Heart Rate Variability as an Indicator of Autonomic Nervous System Disturbance in Tetanus

**DOI:** 10.4269/ajtmh.19-0720

**Published:** 2019-12-12

**Authors:** Ha Thi Hai Duong, Girmaw Abebe Tadesse, Phung Tran Huy Nhat, Nguyen Van Hao, John Prince, Tran Duc Duong, Trịnh Trung Kien, Le Thanh Hoang Nhat, Le Van Tan, Chris Pugh, Huynh Thi Loan, Nguyen Van Vinh Chau, Lam Minh Yen, Tingting Zhu, David Clifton, Louise Thwaites

**Affiliations:** 1 Hospital for Tropical Diseases, Ho Chi Minh City, Vietnam;; 2 Institute of Biomedical Engineering, University of Oxford, Oxford, United Kingdom;; 3 Oxford University Clinical Research Unit, Ho Chi Minh City, Vietnam;; 4 University of Medicine and Pharmacy, Ho Chi Minh City, Vietnam;; 5 Nuffield Department of Medicine, University of Oxford, Oxford, United Kingdom;; 6 Centre for Tropical Medicine and Global Health, University of Oxford, Oxford, United Kingdom

## Abstract

Autonomic nervous system dysfunction (ANSD) is a significant cause of mortality in tetanus. Currently, diagnosis relies on nonspecific clinical signs. Heart rate variability (HRV) may indicate underlying autonomic nervous system activity and represents a potentially valuable noninvasive tool for ANSD diagnosis in tetanus. HRV was measured from three 5-minute electrocardiogram recordings during a 24-hour period in a cohort of patients with severe tetanus, all receiving mechanical ventilation. HRV measurements from all subjects—five with ANSD (Ablett Grade 4) and four patients without ANSD (Ablett Grade 3)—showed HRV was lower than reported ranges for healthy individuals. Comparing different severities of tetanus, raw data for both time and frequency measurements of HRV were reduced in those with ANSD compared with those without. Differences were statistically significant in all except root mean square SD, indicating HRV may be a valuable tool in ANSD diagnosis.

Tetanus is a severe disease characterized by toxin-mediated disinhibition of autonomic and motor nervous systems. Motor neuron disinhibition causes characteristic muscle spasms, whereas autonomic nervous system disinhibition results in fluctuating blood pressure, tachycardia, and pyrexia. When mechanical ventilation is available, spasms can be controlled, but autonomic nervous system dysfunction (ANSD) remains a principal cause of mortality.^1,2^ Robust methods of detecting ANSD suitable for implementation in resource-limited settings where most tetanus occurs would allow earlier intervention and may improve outcome. Diagnosis is currently based on nonspecific clinical signs of pyrexia, sweating, and increased or fluctuating heart rate and blood pressure.^3^ Other methods include 24-hour collections of urinary catecholamines, but this has low specificity, unsuitable for routine use.^4^

In health, heart rate is carefully controlled by the autonomic nervous system. Alterations in parasympathetic and sympathetic nervous system activity result in beat-to-beat heart rate variation, and hence, this variation (heart rate variability [HRV]) reflects autonomic nervous system activity. Heart rate variability is altered in pathological states, such as ischemic heart disease, and reduced variability is predictive of worse outcomes.^5^ Standardized measures of HRV can be calculated from electrocardiogram (ECG) R–R intervals, and consensus guidelines on appropriate indicators are available.^5^ Time domain variables are calculated directly from R–R intervals (termed normal-to-normal intervals), for example, SD. Frequency domain variables are generated from ECG spectral analysis, usually following fast Fourier transformation.^5^ By observing changes in these components after administering autonomic nervous system antagonists, relative contributions of parasympathetic and sympathetic nervous systems have been inferred. Whereas total power of the spectrum represents the general level of autonomic activation, low-frequency activity (< 0.15 Hz) is mainly due to baroreceptor reflex modulation and related to both vagal and sympathetic influence, and high-frequency activity is mainly aligned with vagal activity. Low to high frequency ratio is accepted to indicate the balance between both systems; however, this interpretation fails to take account of effects such as different temporal patterns of sympathetic and parasympathetic components and cardiac pacemaker sensitivity.

Heart rate variability changes in tetanus are largely unknown. Sykora et al.^6^ analyzed baroreflex sensitivity and time domain variables in an 87-year-old woman with tetanus and reported decreased baroreceptor sensitivity compared with a control of similar age; however, the patient, but not control, received mechanical ventilation and a beta-blocker, both of which can influence sensitivity. Goto et al.^7^ reported reduced frequency domain variables in an 11-year-old child; however, this recording was taken following a cardiac arrest and on the 122nd day of hospitalization, when clinical recovery from tetanus is normally expected.

Nevertheless, ANSD diagnosis and prognostication through HRV remains an attractive prospect because of its noninvasive nature. Hitherto, required monitoring equipment was rarely available in settings where most tetanus occurs, but growing availability of low-cost sensors means measurement is increasingly feasible in low-resource settings.^8^ In this study, we aim to investigate the relationship of HRV and ANSD in patients with severe tetanus, providing proof-of-principle that such monitoring may be valuable.

The study was conducted in the Intensive Care Unit at the Hospital for Tropical Diseases, Ho Chi Minh City, between October 2016 and January 2017 and was approved by the Ethical Committee of the Hospital for Tropical Diseases. Written informed consent was given by all participants or representatives before enrollment.

Adults with severe tetanus (Ablett Grade 3 or 4) diagnosed according to the Hospital for Tropical Disease guidelines^9,10^ and receiving mechanical ventilation were recruited to the study. Recruitment was pragmatic and depended on availability of suitable monitors. Ablett Grade 3 was defined as “severe spasms interfering with breathing” and Grade 4 as Grade 3 but with ANSD.^10^ Autonomic nervous system dysfunction was diagnosed clinically by the attending physician but required the presence of at least three of the following within 12 hours: heart rate > 100 bpm, systolic blood pressure > 140 mmHg, blood pressure fluctuation with minimum mean arterial pressure < 60 mmHg, and temperature > 38°C without evidence of intercurrent infections.

Tetanus management followed a standard protocol previously described,^11^ consisting of antibiotics, and spasm control using benzodiazepines and pipecuronium. Autonomic nervous system dysfunction was managed principally with magnesium sulfate.

Electrocardiogram data were collected from bedside monitors (Datex; Datex Ohmeda Inc., GE Healthcare, Helsinki, Finland) in supine undisturbed patients using VSCapture software.^12^ Electrocardiogram, physiological, and clinical data were collected over a 24-hour period. Heart rate variability features were extracted from noise-free 5-minute recordings at 6 am, 12 noon, and 6 pm to prevent bias from HRV diurnal variation.^13^ Time domain variables measured were square root of the mean squared differences of successive normal-to-normal intervals (RMSSD) and SD of all normal-to-normal intervals (SDNN). Frequency domain variables were total power, high-frequency power (0.15–0.4 Hz), low-frequency power (0.05–0.15 Hz), low-frequency normalized units, high-frequency normalized units, and low- to high-frequency ratio. Statistical analyses were performed using R statistical software version 3.5.1 (R Corporation, Vienna, Austria). Data are presented as mean (SD). Heart rate variability was compared between two groups of tetanus severity using a linear mixed-effects model to correct for repeated measurements. A *P*-value < 0.05 was considered statistically significant.

Five patients with Ablett Grade 4 and 5 patients with Ablett Grade 3 tetanus were recruited to the study. Data from one patient with Grade 3 tetanus was too noisy for analysis and, therefore, excluded. Clinical characteristics of the remaining nine patients are given in Table 1. Of these, 8/9 had three high-quality noise-free 5-minute segments at the chosen time point. One patient with Ablett Grade 4 had only two suitable 5-minute segments at 12 noon and 6 pm.

**Table 1:** Clinical data of patients

	Ablett Grade 3,* *n* = 4	Ablett Grade 4* (ANSD), *n* = 5
Age (years)	51.25 (20.66)	58 (7.68)
Males	4 (100)	4 (80)
Tetanus severity score	5.8 (6.5)	4.8 (4.09)
Mechanical ventilation	4 (100)	5 (100)
In-hospital mortality (%)	0 (0)	0 (0)
Maximum heart rate in the 24-hour study period (bpm)	152.8 (22.34)	210.2 (29.26)
Minimum heart rate in the 24-hour study period (bpm)	59.00 (6.21)	48.20 (10.08)
Mean heart rate in the 24-hour study period (bpm)	94.50 (10.25)	90.00 (20.41)
Mean systolic blood pressure in the 24-hour study period (mmHg)	127.60 (2.27)	159.90 (21.06)
Maximum systolic blood pressure in the 24-hour study period (mmHg)	137.50 (9.57)	217.40 (26.49)
Minimum systolic blood pressure in the 24-hour study period (mmHg)	115.00 (5.77)	107.40 (27.24)
Total dose of diazepam during the 24-hour study period (mg)	30.00 (60)	48.00 (65.72)
Total dose of midazolam during 24-hour study period (mg)	120.00 (80.80)	86.40 (84.17)
Total dose of magnesium sulfate during the 24-hour study period (g)	0 (0)	43.20 (26.29)
Total dose of pipecuronium during the 24-hour study period (mg)	38.40 (7.83)	30.72 (4.29)

ANSD = autonomic nervous system dysfunction. Figures given are mean (SD), except males, mechanical ventilation, and mortality, which are *n* (%).

* Ablett Grade 3: severe tetanus with spasms compromising respiration. Ablett Grade 4 is as Grade 3 but with additional signs of autonomic nervous system dysfunction.

Heart rate variability data are presented in Figure 1 and Table 2. All HRV measurements were very low compared with reported ranges for healthy individuals, with low- to high-frequency ratios being significantly greater.^5^ Comparing different severities of tetanus, both time (RMSSD and SDNN) and frequency (low frequency, high frequency, low-frequency normalized units, and total power) variables were reduced in those with ANSD (Ablett Grade 4) compared with those without. Differences were statistically significant in all except RMSSD (*P* = 0.09). Only high-frequency normalized units and low- to high-frequency ratios showed no difference between groups.

**Figure 1.**
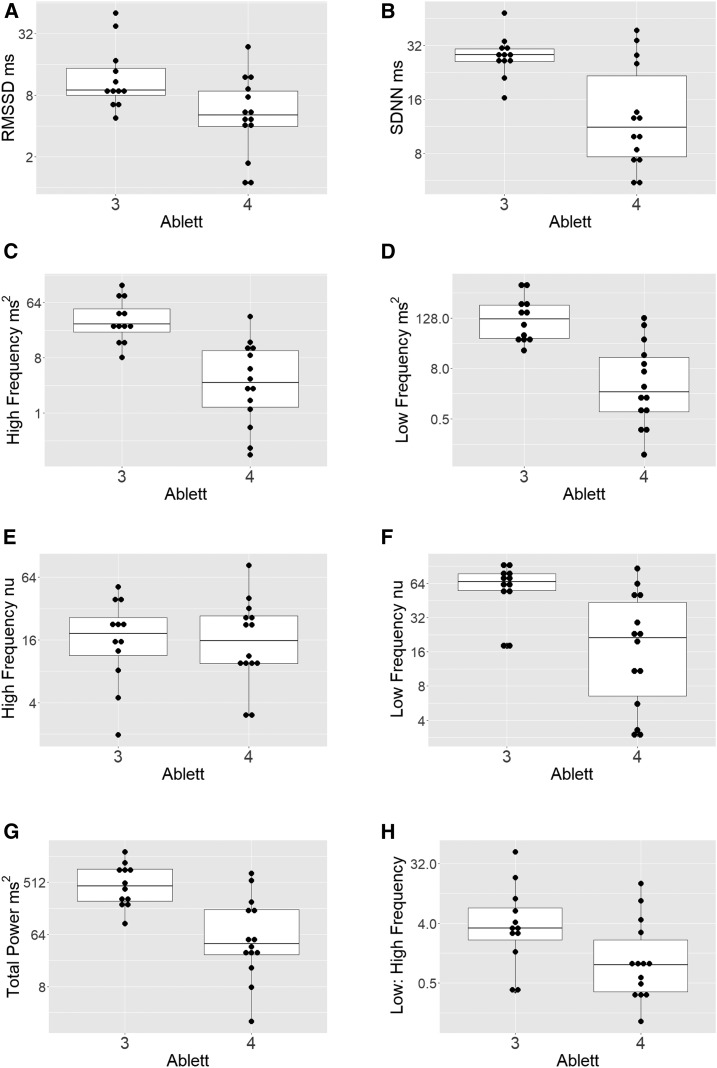
Heart rate variability variables in patients according to Ablett Grade. (**A**) Square root of the mean squared differences of successive normal–normal (NN) intervals (RMSSD), (**B**) SD of all NN intervals (SDNN), (**C**) power in the high-frequency range, (**D**) power in the low-frequency range, (**E**) high-frequency power in normalized units, (**F**) low-frequency power in normalized units, (**G**) total spectral power, and (**H**) low- to high-frequency ratio.

**Table 2:** Heart rate variability variables

	Ablett Grade 3, *n* = 12	Ablett Grade 4 (ANSD), *n* = 14	*P*-value
Root mean square SD (ms)	8.94 (8.03–14.71)	5.13 (3.95–8.86)	0.09
SD of all normal-to-normal (ms)	28.4 (26.03–30.55)	11.23 (7.67–22.40)	0.008
Low frequency (ms^2^)	127.94 (42.37–262.09)	2.29 (0.76–14.95)	0.006
High frequency (ms^2^)	28.80 (21.65–52.81)	3.17 (1.28–10.54)	0.004
Low frequency (normalized units)	66.08 (55.11–77.74)	21.29 (6.86–44.60)	0.04
High frequency (normalized units)	18.72 (11.48–26.80)	16.53 (9.49–27.16)	0.97
Total power (ms^2^)	447.4 (243.3–867.6)	44.46 (28.28–172.22)	0.01
Low- to high-frequency ratio	3.41 (2.32–7.01)	0.94 (0.37–2.44)	0.17

ANSD = autonomic nervous system dysfunction. Variables presented are median (interquartile range). *P*-values are derived from the mixed-effects model.

We present, to our knowledge, the first HRV measurements in a series of patients with tetanus. Our data show a consistent reduction in time and frequency domain variables compared with values reported in healthy subjects. These are particularly reduced in those with clinical signs of ANSD. This is consistent with HRV reported in other pathological states with high levels of sympathetic activation and with existing understanding of ANSD in tetanus.

Sympathetic activation in tetanus is associated with increased circulating catecholamines, which are increased in proportion to disease severity.^4^ These may exert direct effects on the heart and vasculature and indirect effects through reflex reduction in vagal tone. The observed reduction in HRV variables in those with ANSD is consistent with sympathetic nervous system activation. Although the reduction in high-frequency power, suggesting a reduction in vagal tone, is expected, we also observed a reduction in low-frequency power, indicative of both sympathetic and parasympathetic activation. In cases of sympathetic activation, heart rate increases and total power is reduced and, as a result, the low-frequency component may actually decrease.^14^ Similarly at high levels of sympathetic stimulation, a “ceiling effect” may occur at the sinoatrial node when further response cannot occur.^14^

A significant limitation to interpretation of our data is that our patients were all receiving sedative drugs which may influence HRV. Although sedation is not reported to affect HRV in critically ill patients^15^ and subjects in both groups received similar sedative doses, it is possible that drugs were titrated against clinical effect. Magnesium sulfate was used almost exclusively in those with ANSD. Although we have previously shown its use in tetanus is associated with a reduction in urinary catecholamine excretion,^16^ limited data in myocardial infarction suggest that it has limited effect on HRV.^17^

A further limitation is that we used 5-minute recordings to measure time domain variables. Guidelines recommend that these should be measured from 24-hour recordings.^5,14^ Nevertheless, our values are lower than reported 5-minute “normal” values, and our primary comparison was between severity groups.^5^

Heart rate control in tetanus is undoubtedly complex and influenced by many factors not measured in this study. As such, we aimed only to demonstrate that variability is related to disease severity and that alterations in HRV may aid ANSD diagnosis in patients with tetanus. Currently, ANSD diagnosis is limited by poor specificity and may be difficult to distinguish from other causes of cardiovascular instability, such as infection, ischemia, or pain. Heart rate variability could, therefore, potentially be a more sensitive and specific way of identifying those with ANSD. The low HRV observed even in patients with Grade 3 tetanus may represent a clinically less apparent category of ANSD, but where, nevertheless, intervention may be beneficial. Furthermore, HRV changes may be early predictors of subsequent ANSD and enable earlier intervention.

This article has focused on using established HRV measures; however, it is likely these are relatively blunt tools with which to decipher the complex underlying physiological mechanisms and rely on high-quality signals difficult to obtain in critically ill populations in resource-limited settings. However, it may be that obtaining high-quality data becomes more feasible through either increased availability of wearable devices or adaptation of existing equipment.^18^ As newer innovative methods for analyzing data are developed, for example, artificial intelligence, more sensitive ways of analysis could be developed, providing better insight into control mechanisms and disease pathophysiology.

## Supplemental file

Supplemental table
