# Supplementary material for: Heart Rate Variability as an Indicator of Autonomic Nervous System Disturbance in Tetanus
Source: Am J Trop Med Hyg. 2019 Dec 12;102(2):403–7. doi: 10.4269/ajtmh.19-0720 (PMC7008337; doi:10.4269/ajtmh.19-0720)
Supplement: Supplementary file 1 [file tpmd190720.SD1.pdf]

The following are supplemental materials and will be published online only

Supplementary Table: Mixed effects model

Feature	Difference	95% Confidence intervals	p
**Log_2 RMSSD (ms)**	-1.21	-2.58; 0.16	0.09
**Log_2 SDNN (ms)**	-1.15	-2.00; -0.31	0.008
**Log_2 Low Frequency (ms^2)**	-5.08	-8.66; -1.49	0.006
**Log_2 High Frequency (ms^2)**	-3.45	-5.79; -1.11	0.004
**Log_2 Low Frequency normalized units**	-1.68	-3.28; -0.07	004
**Log_2 High Frequency normalized units**	-0.025	-1.20; 1.15	0.97
**Log_2 Total Power (ms^2)**	-3.07	-5.40; -0.75	0.01
**Log_2 Low:Frequency**	-1.66	-3.99; 0.69	0.17
